# Stroke-related health problems and associated actions identified with the post-stroke checklist among nursing home residents

**DOI:** 10.1186/s12872-022-02466-3

**Published:** 2022-02-14

**Authors:** Emma K. Kjörk, Martha Gustavsson, Nohad El-Manzalawy, Katharina S. Sunnerhagen

**Affiliations:** Institute of Neuroscience and Physiology, Department of Clinical Neuroscience, Sahlgrenska Academy at University of Gothenburg, Per Dubbsgatan 14, fl. 3, 413 45 Gothenburg, Sweden

**Keywords:** Nursing home, Long-term care, Follow-up, Stroke, Rehabilitation

## Abstract

**Background:**

Little is known about the needs of permanent nursing home residents after a stroke; comprehensive descriptions of needs are rare. The Post-Stroke Checklist facilitates the identification of health problems. The study aimed to use the Post-Stroke Checklist to identify the extent of health problems, and how they were addressed, in nursing home residents that experienced strokes in Sweden. We also investigated the feasibility of the Checklist in a nursing home context.

**Methods:**

This is a cross-sectional explorative study. Twenty nursing homes in two regions of Sweden participated. We included residents that had experienced a stroke within approximately 3 years and the responsible staff members were approached. Questionnaires were completed during face-to-face meetings with staff members (n = 45) knowledgeable about the residents. Data collection included the Post-Stroke Checklist, Barthel Index, modified Rankin Scale, resident and staff characteristics, and a satisfaction-questionnaire completed by staff.

**Results:**

At the included nursing homes 1061 residents, 22% (n = 239) had a history of stroke, and 6% (n = 65) had experienced strokes during the last 3.5 years. Forty-nine residents were included (41% men, median age, 86 years, range 59–97). Among the health problems identified with the Checklist, activities of daily living (82%) were most common, and spasticity (41%) and pain (29%) were least common. Residents had extensive care needs, with a median of six health problems per resident. The total number of health problems addressed by previous actions i.e., referrals, as suggested in the Checklist, was 124, when recalled by staff. The median Barthel index score was 35. Lack of follow-up after stroke (e.g., by using a checklist) was reported in 17/20 nursing homes. The staff were satisfied with the Post-Stroke Checklist.

**Conclusions:**

We found that more than 1/5 of residents had experienced a stroke; thus, the Post-Stroke Checklist was a useful tool in nursing homes. Half of the residents had more than six health problems, identified with the Post-Stroke Checklist. Extensive needs, combined with a lack of follow-up, indicated a risk of insufficient care. These findings suggested that nursing home routines could be improved with the Post-Stroke Checklist.

*Trial registration* The project is registered in Research web, project number: 256021.

## Introduction

Stroke is a major cause of disability worldwide. With an aging population, more people survive strokes with long-term consequences [1, 2]. In Sweden, after a stroke, 22% of patients are discharged to residential care, including short-stay homes [3]. For those unable to return home, a permanent nursing home is an alternative, which includes round-the-clock care with qualified nurses. Nursing home residents that experience a stroke have high-level needs [4] and represent an aging, frail population [5, 6] with multiple comorbidities [7, 8]. In addition, medical complications after strokes, including pain [9] and neuropsychiatric disorders, can negatively affect recovery [10]. In this clinically complex group, residents often depend on staff members to identify their needs [11] and stroke-specific follow-ups are often lacking [12].

Comprehensive assessments with a multidisciplinary approach are essential to identify care needs in frail, aged individuals [5] and to prevent recurrent stroke and improve functional outcomes and health-related quality of life after a stroke [13]. The Action Plan for Stroke in Europe [14] and the National guidelines for stroke in Sweden recommend performing structured reviews of patients within 3–6 months after a stroke, and regular reviews thereafter. The use of the 11-item Post-Stroke Checklist (Checklist) [15, 16] might benefit residents and assist nursing home staff in identifying health problems and providing guidance.

Research in nursing homes is aggravated by several factors, [17] and studies often exclude individuals with severe stroke and premorbid disabilities [18, 19]. Hence, most stroke research may not be generalisable to nursing home populations. Recent studies have shown that individuals in nursing homes have stroke-related health problems, [20, 21] require high levels of care, [4, 22, 23] and often receive insufficient stroke-care, due to lack of knowledge [24]. However, many previous studies were based on data recorded close to admission, or they did not comprehensively describe long-term resident needs.

The Post-Stroke Checklist was shown to be feasible for individuals living in their own homes; [15, 16] less is known about using the Checklist in nursing homes. The present study aimed to use the Post-Stroke Checklist to identify the extent of health problems, and how they were addressed, in nursing home residents that experienced strokes in Sweden. Additionally, we investigated the feasibility of the Checklist in a nursing home context.

## Methods

### Design, setting, and participants

This cross-sectional explorative study included individuals that had experienced a stroke and lived in nursing homes in two geographic regions of Sweden, between 2019 and 2020.

In Sweden, the need for nursing home care is means-tested, and the costs are heavily subsidised with taxes. Nursing homes always provide qualified nurses and access to physiotherapy and occupational therapy. The rehabilitation staff often conduct assessments at admission.

Here, we identified nursing homes from the national list of nursing homes. We included institutions that varied in size, type of care, geographic location, and socioeconomic environment. We included only permanent nursing homes with more than ten beds. We excluded short-stay nursing homes, available for people when expected to return to their own home e.g., after adaptations. For study eligibility, residents had to have experienced a stroke (reported by a staff nurse) within approximately 3 years prior to study enrolment. Staff members were included when they were involved in the continuous care of a resident included.

This study was approved by the Regional Ethics Review Board in Gothenburg (no. 219–18). Informed written consent was provided by all participants, or a family member, when appropriate.

### Procedure and data collection

#### Procedure

**Fig. 1:**
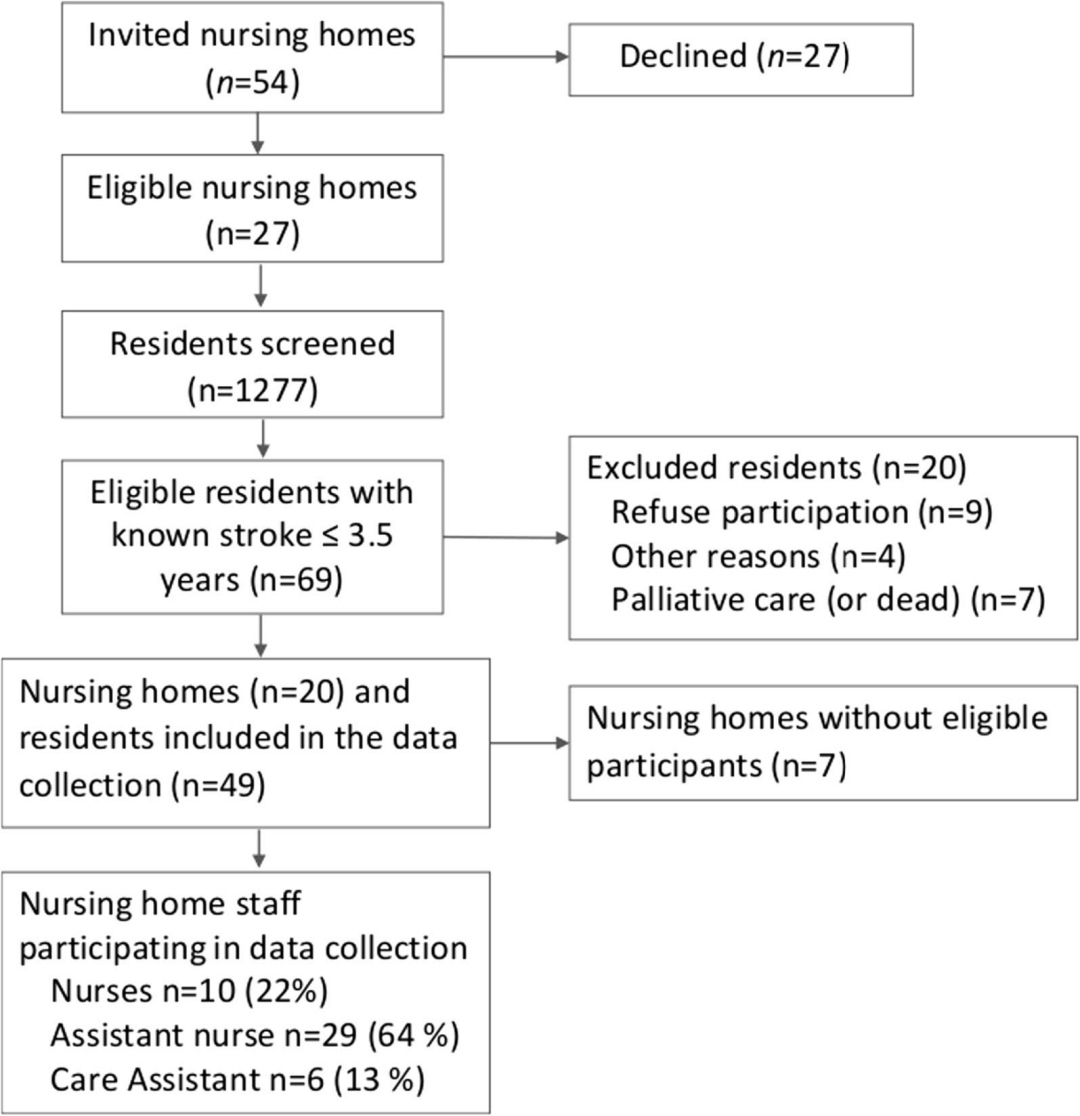
Flow chart of the recruitment process

Nursing home unit managers received study information through email, followed by a phone call from the researchers (EK, MG) to invite them to participate in the study. The recruitment process is shown in the flowchart (Fig. 1). After receiving study information, the responsible nurses reported the number of residents with a known history of stroke, based on their knowledge and/or nurse medical records. Residents that had experienced a stroke within approximately 3 years were asked to participate by the care staff. The diagnosis was later checked, based on hospital medical records.

The researchers received contact information for care staff knowledgeable about the residents. A study information letter and the Post-Stroke Checklist were sent to the care staff in advance. Staff members in the nursing homes were encouraged to discuss the Checklist items with the resident or family member before the data collection meeting, when possible. All data were collected at the nursing home during a face-to-face meeting with the staff and one of the researchers. The researchers completed the assessments and study forms based on the staff´s perceptions of the residents and if they could recall if they had received previous actions in regard to the Checklist.

#### Data collection

The Post-Stroke Checklist [15] was used to identify stroke-related health problems in residents. The Checklist was developed to evaluate items that could impact quality of life and the occurrence of evidence-based interventions, and it was previously shown to be feasible for follow-up [15, 16]. Checklist items are related to secondary prevention, activities of daily living (ADLs), mobility, spasticity, pain, incontinence, communication, mood, cognition, life after stroke, and relationships with family members. The yes/no responses related to each item in the Checklist are followed by recommendations for appropriate actions; e.g., assessment by a physician with knowledge about post-stroke pain [15]. In this study, in addition to the yes/no responses after each item, “don’t know” was added as an extra response category to capture staff uncertainty, since the staff’s knowledge about residents’ could be limited. In addition, after the recommended actions related to each item, response options (yes/no/don’t know) were added, to indicate whether previous referrals, assessments or care had been taken prior to the use of the Checklist in current study (e.g,. have previous referrals to specialist Speech and Language Therapist for further assessment taken place before this study?).

The Barthel Index (BI) [25] was used to assess independence in personal self-care. Scores ranged from 0 to 100, where 100 indicated independence in self-care. Modified Rankin Scale (mRS) [26] was used to measure the degree of disability. Scores ranged from 0 to 5, where 0 indicated no significant disability, and 5 indicated severe disability.

Nursing home and resident characteristics (demographics and clinical) were collected with a study form and later confirmed and complemented by hospital medical records. Most items had yes/no responses. Two items were multiple-choice responses, and two items had open responses that were dichotomised in the analysis. Space for additional comments was available. We retrieved data from hospital medical records on stroke-specific factors, the National Institutes of Health Stroke Scale, [27] comorbidity, activity level at discharge, and personal factors. In addition, a questionnaire was administered to evaluate staff satisfaction with the Post-Stroke Checklist, including questions published in previous studies [15, 16]. The responses were rated on a Likert scale of 1 to 5, where 1 indicated not satisfied and 5 indicated completely satisfied. We also recorded background data on the staff (profession, sex, country of birth, working years, stroke expertise/education, number of residents assessed with the Checklist). In addition, a multiple-choice question was included to determine whether the staff felt that any of the following items were missing from the Checklist: nutrition/swallowing, oral care, fatigue, sex, irritability, personality, work, social relations, or attention.

### Analysis

Data collected from the Checklist and clinical assessments are expressed on an ordinal scale, and they were analysed with non-parametric statistics. Data were analysed with descriptive statistics with SPSS version 24 (Statistical Package for the Social Sciences Inc.Armonk, NY: IBM Corp).

## Results

### Nursing home characteristics

Ten nursing homes (50%) had more than 50 beds (range 16–108) in total. These large homes were organised in sub-sections, with a median of 11 beds each. Eleven nursing homes were located in urban areas and nine were located in rural areas. Three nursing homes were privately operated, but included in the subsidised modelled. All nursing homes had facilities for common activities (median: 14 activities, range 0–100 scheduled activities per month), and seven had a designated room for physical training. An occupational therapy assistant was available in half of the nursing homes to support scheduled activities. All nursing homes had team meetings regularly, where care and rehabilitation staff, including occupational therapist and physiotherapist, discussed the needs of residents. In addition, other communication channels (i.e., telephone) were used for referrals, mostly initiated by the care staff. Seventeen nursing homes lacked follow up after a stroke (i.e., with a checklist), and only one nursing home offered the staff education about stroke, and that was web-based. None of the included care staff had formal stroke expertise (one nurse was taking a stroke course at the university). All nursing homes used written care plans for each resident, including how to carry out daily routines, and the plans were typically updated every 3–6 months.

### Participant characteristics

More than 20% of residents had experienced at least one stroke, and 6% had experienced a stroke within the preceding 3.5 years. Of the 49 residents included, 11 lived in a nursing home focused on dementia compared to a more general focus. All residents had one or more comorbidities, confirmed by the hospital medical records (Table 1). In 23 residents, comorbidities were considered the main reason for limited functioning, rather than the stroke. The BI scores indicated a high level of dependency (median score: 35, IQR 10–70). Dependencies were higher in men (median score: 25, IQR 6–55) compared to women (median score: 40, IQR 15–75). In addition, women had lower mRS scores (median score: 4) than men (median score: 5). Women (median age: 88 years, IQR 85–92) were more than 10 years older than men (median: 79 years, IQR 73–86). One in four (18%) residents had experienced a haemorrhagic stroke, and nearly half of the residents had a previous documented stroke (Table 1). Forty-five nursing home staff members (76% women, 38 born in Sweden) participated. The median time working in a nursing home setting was 15 years (range 0.5–42), and the majority were assistant nurses (n = 29; Fig. 1).

**Table 1:** Participant characteristics mainly based on hospital medical records among residents with stroke ≤ 3.5 years (n = 49)

	Whole sample (n = 49)
Age in years, median (IQR)	86 (79–91)
Months since stroke, median (IQR)	18 (10–34)
Born in Nordic countries, n (%)	44 (90)
Inability to speak the local language n (%)	4 (8)
Pre-stroke living conditions, n (%)	
Nursing home	11 (25)
Assisted care in own home	17 (39)
Stroke type, n (%)	
Confirmed stroke	45 (92)
Ischemic stroke	37 (82)
Stroke location n %	
Right	24 (53)
Left	20 (45)
Bilateral	1 (2)
Stroke severity, n %	
NIHSS^a^ median (IQR)	8 (4–14)
Mild stroke (0–5)	19 (40)
Moderate stroke (6–14)	16 (33)
Severe stroke (15–24)	11 (23)
Very severe stroke (≥ 25)	2 (4)
Stroke-related outcomes n %	
Length of hospital stay in days, median (range)	16 (2–90)
Wheel-chair use at discharge	29 (62)
Swallowing problems	20 (42)
Stroke-related visual impairment	7 (14)
Comorbidities, n (%)	
Previous stroke	23 (47)
Dementia	9 (18)
Cognitive impairment	20 (41)
Participants with documented comorbidities	49 (100)
(Included, cardiovascular, diabetes and cancer)	
Discharge destination from hospital n (%)	
Own home	10 (20)
Short-stay nursing home	22 (45)
Permanent nursing home	16 (33)

^a^National Institutes of Health Stroke Scale (NIHSS) measured ≤ 24 h of admission, normal values 0–42. Missing data: Data on stroke type and location (n = 4), NIHSS (n = 1), months since stroke (n = 1), pre-stroke living condition (n = 5), wheelchair use (n = 2, discharge destination (n = 1). Values are presented as numbers and valid percentages unless stated otherwise

### Stroke-related health problems and actions identified with the Checklist

**Fig. 2:**
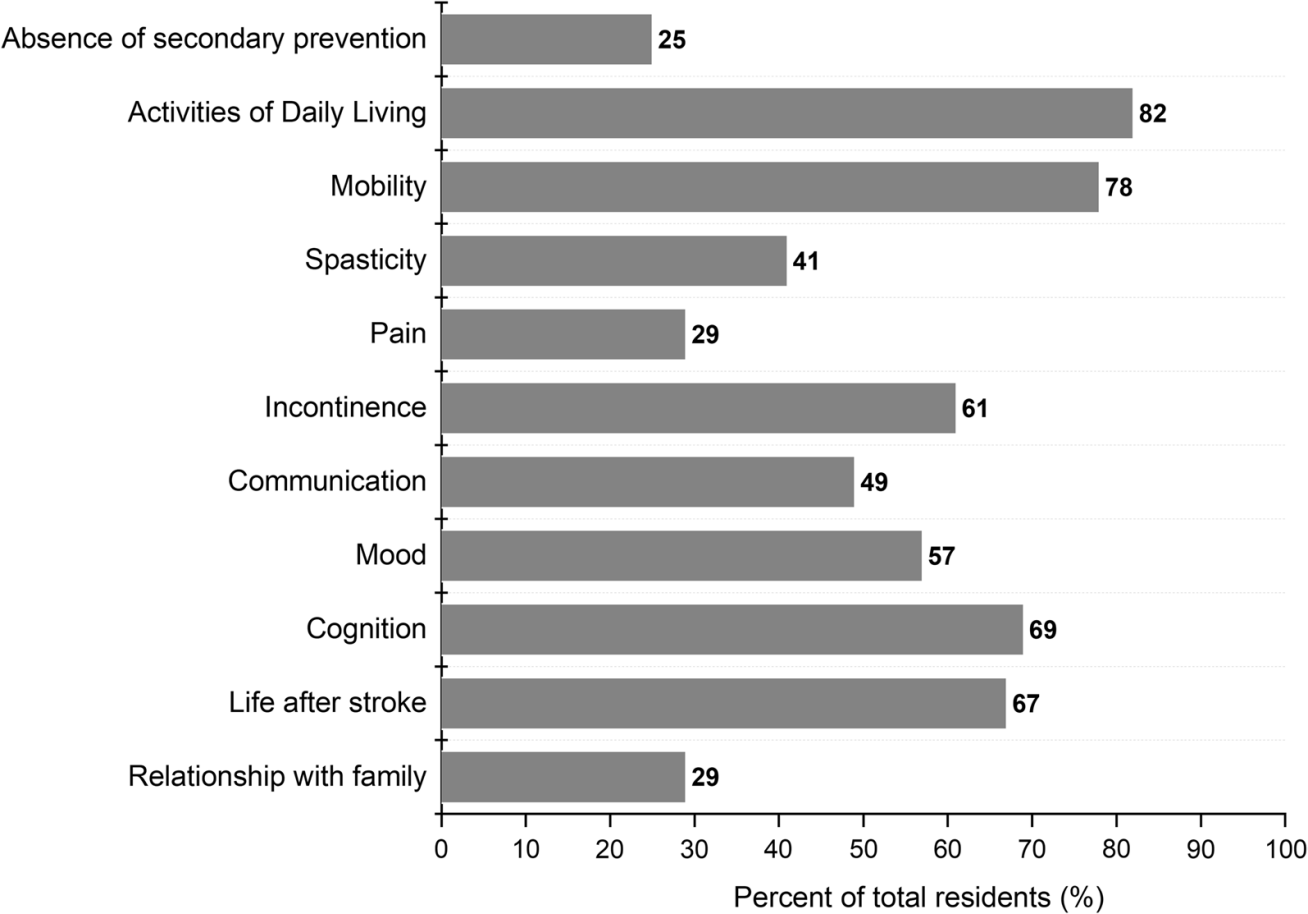
Proportions of residents with identified stroke related health problems in each post-stroke checklist item (n = 49)

**Fig. 3:**
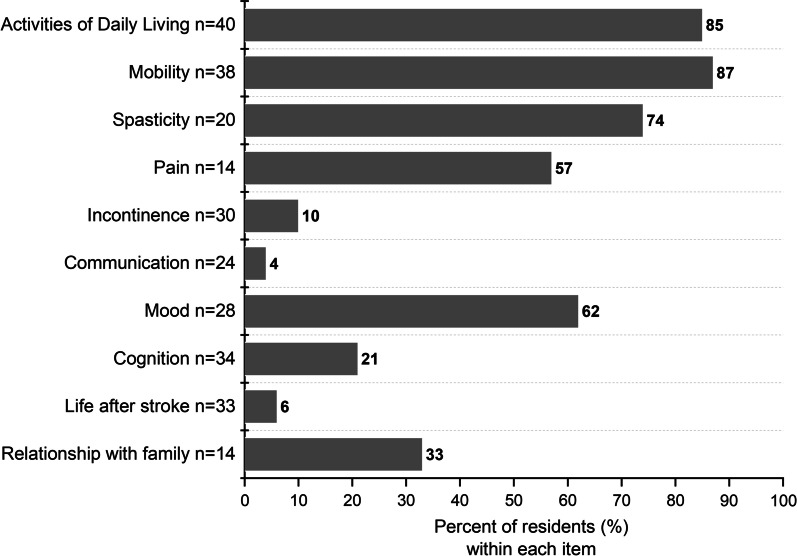
Percentages of residents with received actions targeting the health problems identified by the post-stroke checklist including assessments and referrals

All except one resident had at least one health problem identified with the Checklist (secondary prevention excluded). The median was six problems per resident (IQR 4–8; Fig. 2). Most residents with identified problems in ADL (n = 40) and mobility (n = 38) were assessed by rehabilitation professionals (Fig. 3). In contrast, only one of 24 residents with communication problems was referred to a speech therapist. Furthermore, 6 of 14 residents with identified pain had not received actions related to their stroke. In addition to referrals and assessments (Fig. 3), 30 residents had received one or more interventions related to a Checklist item, based on reports from staff members.

### Usefulness of the Checklist

Of 45 staff members, 16 administered the Post-Stroke Checklist in dialogue with residents or the next of kin, before the data collection meeting together with the researcher. Staff members used the Checklist for a median of one resident each (range: 1–4); 34 staff members perceived they had sufficient knowledge to use the Checklist. Most staff members (90%) were satisfied or very satisfied with the ability of the Checklist to identify health problems after a stroke; 87% of staff members would absolutely recommend using the Checklist in nursing homes (Table 2).

**Table 2:** Evaluation of the use of Post-Stroke Checklist (PSC) based on satisfaction ratings (Likert 1–5) by nursing home staff (n = 45), 1 indicated not satisfied and 5 completely satisfied

	Median (IQR^a^)
General use	4 (4–5)
Identification of needs	5 (4–5)
Guidance for referrals and treatment	4 (4–5)
Recommend using PSC in nursing homes (n = 44)	5 (4–5)

^a^Interquartile range (IQR)

When asked about whether the Checklist missed any areas for assessing residents, only five staff members answered yes. Furthermore, when asked about missing specific areas, 17 answered no, and 28 answered yes; the following areas were missing: nutrition (n = 18), fatigue (n = 12), irritability (n = 10), oral care (n = 7), social relations (n = 6), personality (n = 4), vision (n = 2), attention (n = 1), and sex (n = 1).

## Discussion

The Post-Stroke Checklist identified a number of stroke-related health problems, which were not always met with appropriate actions. The lack of follow-up routines, insufficient knowledge about stroke, and inadequate actions should be addressed in nursing homes. Considering the staff’s high satisfaction with the Checklist, it would be feasible for use in nursing homes as a first step towards improving care.

The residents in this study had complex needs associated with a wide range of health problems combined with frailty. The proportion having had haemorrhagic strokes (18%) in our study is a bit more than the average in Sweden (around 10%) [3] and shows that these who survive often are more dependent and thereby need nursing home care. Recently, the proportion of older individuals living in nursing homes has decreased in Sweden; [3] hence, only those that need the highest levels of care are moving to nursing homes, similar to trends in the UK [4]. Consequently, nursing home staff must handle complex diseases, and stroke rehabilitation is not the primary focus. Thus, there is a risk that rehabilitation needs will not be met, [14, 28] particularly when the time allotted for in-patient rehabilitation is limited. Moreover, consistent with our findings, other studies have shown that, after a severe stroke, the main focus may not be to improve function, but to reduce complications, [19] often within an end-of-life care perspective [28]. Consequently, rehabilitation includes the equipment and adjustments used in clinically complex situations. Nevertheless, residents may experience improvements in function, and all residents should receive rehabilitation according to their needs [29].

We found that residents that required post-stroke care had a median of six health problems each, based on the Post-Stroke Checklist. This result illustrated the need for a multi-dimensional follow-up. Previous studies in community-dwelling populations reported a median of 3–4 problems per resident [15, 16]. Not surprisingly, the most common health problems we identified were ADLs (82%), which was confirmed by the high level of dependency we observed, consistent with previous studies [22]. We also identified pain (29%) and mood problems (57%) among residents, consistent with previous studies [20, 23]. The deterioration in ADL after the subacute phase, [30] combined with the fact that residents are often inactive in nursing homes [31] highlighted the importance of identifying long-term needs.

The identified health problems were not addressed, or at least staff did not know if residents had received any previous referrals, assessments, or care according to the suggested actions in the Checklist. For example, six of 14 residents that reported pain were not assessed for pain in a post-stroke assessment. Research has shown that, when stroke-specific care was insufficient in nursing homes, [24] treatable symptoms might not be addressed. In the present study, actions within the expertise of staff at the nursing home were performed more often than, for example, communication therapy, because speech therapists were lacking in the team. Furthermore, there is a gap between the need for communication therapy and available speech therapists in primary and community care [12]. Moreover, occupational therapists and physiotherapists mainly performed assessments of rehabilitation needs close to admission, for all residents. Consequently, care staff members were expected to detect subsequent health problems and communicate them to the responsible physician or rehabilitation team for appropriate actions.

A structured follow-up (e.g., by using a stroke related checklist) was not provided in most (n = 17/20) nursing homes, consistent with previous research [22] and national surveys [12]. Currently, post-stroke follow-ups are not equitable, because individuals in nursing homes are often excluded [12]. Nevertheless, the importance of follow-up [14] is underlined by our finding that 22% of residents in nursing homes had a history of stroke, consistent with previous studies (range 16–25%) [22, 24]. Residents are most likely to benefit from post-stroke follow-ups performed in the nursing home setting, because a change of environment can have negative effects. Thus, we suggest the Post stroke checklist could be used at the nursing home environment where a nurse is responsible, and each resident have a physician as well.

The staff members’ satisfaction with the Checklist was high; three in four staff members perceived that they had sufficient knowledge to conduct the assessment. Checklist use in this study was challenged by the lack of stroke education among staff members, and the inability of residents to give their own perceptions. Despite these challenges, the Checklist could empower residents that have difficulty communicating their needs by providing them with a more equitable follow-up. As stated previously, the Checklist should be complemented with dialogue [16] to ensure all health problems are identified. However, because the Checklist was primarily developed for use in primary care [15], it might require adjusting to suit a nursing home context. Indeed, some areas important to nursing home residents were missed in this study; for example, nutrition and oral care. This finding was confirmed by the finding that 42% of the residents had swallowing difficulties at discharge. Therefore, it could be relevant to add items to the Checklist that pertain to the nursing home context, particularly because stroke-care experience varied among staff members. Only one nursing home in this study offered stroke-specific education for staff members. Uncertainty about stroke care among staff members was raised in a previous study [24]. By providing education (i.e., the national web-based education) and implementing the Checklist, the level of care for older individuals that experience a stroke could be improved.

### Strengths and limitations

The main strength of this study was the naturalistic design and the representative group of nursing home residents. Our findings illustrated a broad range of stroke-related problems within a group of individuals often excluded from research. We endeavoured to obtain the best available data for residents that often have difficulties communicating their needs.

This study had several limitations. First, the sample size was small, due to the comprehensive study logistics and Covid-19 restrictions on the number of participants allowed. To overcome challenges in nursing home research, we sought early involvement by staff and a flexible approach to facilitate recruitment and data collection [17]. Nevertheless, several nursing homes declined to participate. However, the included nursing homes represented a variety of geographic areas where some areas represent people such as immigrants and refugees that are particularly vulnerable groups. Although we aimed to screen all residents that had experienced a stroke, a selection bias could not be ruled out, because the data were collected retrospectively from nurses. It would also have been valuable to have more clinical information regarding those not included. Second, the Checklist was completed with limited input from the residents. This method was applied due to the frailty of individuals in this group. Consequently, because staff members might not have been fully aware of the prevalence of some health problems, a recall bias could not be excluded. Notably, staff members were encouraged to ask residents or a family member to validate the responses in the Checklist. Moreover, we adapted the instruments [17] by adding an “I don’t know” alternative to capture the lack of knowledge. Third, staff members might have had difficulty judging whether some problems were stroke related. Therefore, the comorbidities and the variety of stroke experience among staff members constituted confounding factors. Nevertheless, the key message was that the Checklist successfully identified common health problems in this population that could be addressed with targeted interventions.

Finally, this study contributed new knowledge that could be of interest internationally. However, our results should be interpreted with caution because nursing homes differ widely among different countries. Future studies are needed with a broader representation that involves other regions and individuals that receive comprehensive care in their own homes.

## Conclusion and implications

We concluded that the Post-Stroke Checklist could support care staff members in identifying the needs of residents. Overall, the conditions observed in the nursing homes in this study (i.e., team-meetings, access to rehabilitation professionals, and care plans) provided a good basis for implementing a multidisciplinary follow-up with the Post-Stroke Checklist. In this study, a large proportion of residents (> 20%) had experienced a stroke. The Checklist showed that half of the included residents had more than six health problems each. This high level of needs, combined with a lack of structured follow-ups, and a lack of stroke education among staff members, constituted a risk of insufficient stroke care. These findings suggested that follow-up routines in nursing home settings could be improved quite readily by using the Post-Stroke Checklist.

## Data Availability

Complete data cannot be made public due to Swedish regulations (https://etikprovning.se/for-forskare/ansvar/). The data can be available upon reasonable request to the authors. Please contact Professor Katharina S. Sunnerhagen, ks.sunnerhagen@neuro.gu.se.

## Abbreviations

Checklist: The Post-Stroke Checklist
BI: The Barthel Index
mRS: The modified Rankin Scale
NIHSS: The National Institutes of Health Stroke Scale
ADL: Activities of Daily Living
